# Supplementary material for: Content-rich biological network constructed by mining PubMed abstracts
Source: BMC Bioinformatics. 2004 Oct 8;5:147. doi: 10.1186/1471-2105-5-147 (PMC528731; doi:10.1186/1471-2105-5-147)
Supplement: Additional File 5 — The original Chilibot query results of the term "long-term potentiation (LTP)" and 22 other terms, limiting the latest references analyzed to the years 1990, 1995, 2000, and 2004. [file 1471-2105-5-147-S5.bz2 › chilibotAdditionalFile5/ltp2004/html/LTP_ACTININ.html]

 


 **LTP** and **ACTININ** 
  
Found 3 abstracts in PubMed,  **3 abstracts were retrieved and analyzed**.  


---

 Search Google  |
 PDF files only 
|  EDU domain only 

---

- J Biol Chem, 2002   Ca influx through the N methyl d aspartate type glutamate receptor leads to activation and postsynaptic accumulation of Ca calmodulin dependent protein kinase II and ultimately to long term potentiation **[LTP]**, which is thought to be the physiological correlate of learning and memory.
  The NMDA receptor also serves as a CaMKII docking site in dendritic spines with high affinity binding sites located on its NR1 and NR2B subunits.
  We demonstrate that high affinity binding of CaMKII to NR1 requires autophosphorylation of Thr.
  This autophosphorylation reduces the off rate to a level = approximately 23 min that is similar to that observed for dissociation of the T286D mutant CaMKII = approximately 30 min from spines after its glutamate induced accumulation Nat.
  Neurosci.
  81 886 .
  CaMKII as well as the previously identified NR1 binding partners calmodulin and alpha **actinin** bind to the short C terminal portion of the C0 region of NR1.
  Like Ca calmodulin, autophosphorylated CaMKII competes with alpha **actinin** 2 for binding to NR1.
  We conclude that the NR1 C0 region is a key site for recruiting CaMKII to the postsynaptic site, where it may act in concert with calmodulin to modulate the stimulatory role of alpha **actinin** interaction with the NMDA receptor.

  - Neuron, 2001   **A model of synaptic memory a CaMKII PP1 switch that potentiates transmission by organizing an AMPA receptor anchoring assembly.** .
    Ca2ion calmodulin dependent protein kinase II CaMKII is localized in the postsynaptic density PSD and is necessary for **LTP** induction.
    Much has been learned about the autophosphorylation of CaMKII and its dephosphorylation by PSD protein phosphatase 1 PP1 .
    Here, we show how the CaMKII PP1 system could function as an energy efficient, bistable switch that could be activated during **LTP** induction and remain active despite protein turnover.
    We also suggest how recently discovered binding interactions could provide a structural readout mechanism the autophosphorylated state of CaMKII binds tightly to the NMDAR and forms, through CaMKII **actinin** actin 4.1 SAP97 linkages, additional sites for anchoring AMPARs at synapses.
    The proposed model has substantial experimental support and elucidates principles by which a local protein complex could produce stable information storage and readout.

    - Eur J Pharmacol, 1999   The glutamatergic synapse is the key structure in the development of activity dependent synaptic plasticity in the central nervous system.
      The analysis of the complex biochemical mechanisms at the basis of the long term changes in synaptic efficacy have received a tremendous impulse by the observation that the post synaptic constituents of the synapse can be separated and purified through a simple procedure involving detergent treatment of synaptosomes and differential centrifugation.
      In this fraction, called post synaptic density , the functional interactions of its constituents are preserved.
      The various subunits of ionotropic glutamate receptors are held in register with the presynaptic active zone through their interaction with linker proteins.
      N methyl D aspartate subunits NR2A and NR2B, bind to the PSD protein called PSD 95, which in turn binds neuroligins, providing a handle for interacting with neurexin, located in the plasma membrane at the presynaptic active zone.
      Additional clustering of NMDA receptors is provided through the binding of NRI subunits to the cytoskeletal protein alpha **actinin** 2.
      AMPA and kainate receptors are other important constituents of PSDs and bind to different anchoring proteins.
      Phosphorylation processes have long been known to modulate NMDA receptor functional activity the finding that several protein kinases, particularly Ca2ion Calmodulin dependent protein kinase II and protein tyrosine kinases of the src family, are major constituents of PSDs has allowed to demonstrate that these enzymes are localized in a strategic position of the glutamatergic synapse, so that their activation provides a means for NMDA receptor function regulation upon its activation.
      The relevance of these mechanisms has been demonstrated in experimental models of pathologies involving deficits in synaptic plasticity, such as in streptozotocin induced diabetes and in an animal model of prenatal induced ablation of hippocampal neurons.
      Both animal models display disturbances in long term potentiation **[LTP]** and cognitive deficits, thus providing in vivo models to study pathology related changes in both the structure and the function of the excitatory synapse.
